# Bistable Perception Modeled as Competing Stochastic Integrations at Two Levels

**DOI:** 10.1371/journal.pcbi.1000430

**Published:** 2009-07-10

**Authors:** Guido Gigante, Maurizio Mattia, Jochen Braun, Paolo Del Giudice

**Affiliations:** 1 Wolf Soluzioni, Rome, Italy; 2 Istituto Superiore di Sanità, Rome, Italy; 3 INFN, Sezione Roma 1, Rome, Italy; 4 University of Magdeburg, Magdeburg, Germany; University College London, United Kingdom

## Abstract

We propose a novel explanation for bistable perception, namely, the collective dynamics of multiple neural populations that are individually meta-stable. Distributed representations of sensory input and of perceptual state build gradually through noise-driven transitions in these populations, until the competition between alternative representations is resolved by a threshold mechanism. The perpetual repetition of this collective race to threshold renders perception bistable. This collective dynamics – which is largely uncoupled from the time-scales that govern individual populations or neurons – explains many hitherto puzzling observations about bistable perception: the wide range of mean alternation rates exhibited by bistable phenomena, the consistent variability of successive dominance periods, and the stabilizing effect of past perceptual states. It also predicts a number of previously unsuspected relationships between observable quantities characterizing bistable perception. We conclude that bistable perception reflects the collective nature of neural decision making rather than properties of individual populations or neurons.

## Author Summary

The instability of perception is one of the oldest puzzles in neuroscience. When visual stimulation is even slightly ambiguous, perceptual experience fails to stabilize and alternates perpetually between distinct states. The details of this ‘bistable perception’ have been studied extensively for decades. Here we propose that bistable perception reflects the stochastic integration over many meta-stable populations at two levels of neural representation. While previous accounts of bistable perception rely on an oscillatory dynamic, our model is inherently stochastic. We argue that a fluctuation-driven process accounts naturally for key characteristics of bistable perception that have remained puzzling for decades. For example, our model is the first to explain why the statistical variability of successive dominance periods remains essentially the same, while the mean alternation rates of bistable phenomena range over two orders of magnitude. By postulating two levels of representation that are driven by stimulation and by perceptual state, respectively, our model further accounts for the stabilizing influence of past perceptual states, which are particularly evident in intermittent displays. In general, a fluctuation-driven process decouples the collective dynamics of bistable perception from single-neuron properties and predicts a number of hitherto unsuspected relations between behaviorally observable measures.

## Introduction

Certain visual displays are not perceived in a stable way but, from time to time and seemingly spontaneously, their phenomenal appearance wavers and settles in a distinctly different form. This phenomenon is called bistable perception and occurs with a variety of ambiguous visual displays (*e.g.*, [1]), as well as with ambiguous stimuli in the auditory (*e.g.*, [2]) and tactile domains [3]. The most extensively studied instance is binocular rivalry [4]–[7], where the phenomenal experience of an observer alternates between two images that are continuously presented to the left and right eye, respectively. In spite of the somewhat ‘unnatural’ method of stimulus delivery, there is good evidence that binocular rivalry shares the typical properties of other instances of bistable perception [8]–[11].

One typical property of bistable perception is that phenomenal appearance shifts irregularly, so that a particular appearance lasts for varying lengths of time. The average such “dominance time” varies by one or two orders of magnitude (typically seconds to tens of seconds) between individual observers [12],[13] and between different bistable displays [10],[11],[14],[15]. Even for the same observer and same display, dominance times vary substantially with stimulus intensity [16],[17], with attention [18]–[21], and when a display is periodically interrupted [22]–[24]. In some cases, the average dominance time experienced by a given observer on a given display under different stimulus regimes may differ by two orders of magnitude [21].

Another typical property is that the statistical distribution of dominance times is well approximated by a Gamma function [14],[25],[26]. In general, the shape parameter 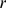 of the Gamma function falls into a surprisingly narrow range with values from 3 to 6 [25]–[30], although values from 2 to 20 have also been reported (*e.g.*, [31]).

Whereas bistable perception was long considered a “memoryless” process [25],[27],[28],[31], it has become clear that phenomenal appearance can be influenced by past perceptual states. For example, when the presentation of an ambiguous display is interrupted and later resumed, the dominant appearance often remains the same [22]–[24]. This persistence of the dominant appearance stabilizes perception considerably, slowing or even arresting perceptual reversals for intermittently presented displays. The ‘memory’ in question reflects a longer history of dominance periods, not merely the last dominance period before the stimulus interruption [32],[33].

It is not known what mechanisms allow a ‘memory’ of perceptual appearance to persist and to influence the appearance of subsequent stimulation. One possibility are adaptation states at the level of perceptual representations, as such states are known to persist over short stimulation gaps and to influence subsequent appearance [32],[34],[35]. Another possible mechanism would be some kind of short-term or working memory at post-perceptual levels of processing [24],[36]. Qualitatively, the effect of ‘memory’ can be summarized as follows: the longer an appearance has dominated perception in the recent past, the more likely it is to dominate perception again. The effect of ‘memory’ is evident for continuous and, more markedly, intermittent stimulation, and appears to be comparatively long-lasting (*i.e.*, minutes rather than seconds [33],[37]).

We propose a model for the dynamics of bistable perception with two novel elements: (i) stochastic integration over multiple meta-stable populations and (ii) two separate levels of representation (sensory information and phenomenal experience). Our central intuition is that perceptual bistability reflects the collective properties of many meta-stable populations rather than specific biophysical properties of single neurons (see also [38]). Together, these two elements account for several hitherto puzzling aspects of bistable perception, including the wide range of time-scales of perceptual alternations, the existence and characteristics of memory effects, the highly conserved shape of dominance distributions, and others. Our model predicts the perceptual dynamics of bistable displays for a variety of stimulation regimes, including continuous and intermittent presentation. Although formulated at the level of abstract populations, our model could readily be extended to a biophysically detailed description of spiking neurons. As our model aims to account for comparatively slow processes (O(10 s)), it neglects phenomena such as fast adaptation.

Several computational accounts for binocular rivalry have been proposed previously. All postulate some form of reciprocal inhibition between two rivaling representations [39]–[43]. Some recent models are biophysically more realistic and are formulated in terms of spiking neurons. In addition to mutual inhibition, these models postulate some form of fast adaptation for the currently dominant population (in the firing rate, the synaptic efficacy, or both), which curtails dominance times and enforces perceptual reversals [44]–[46]. In yet other models, the effect of adaptation is complemented by noise-driven transitions [17], [47]–[49]. Some recent models have introduced an additional form of slow adaptation in order to account for memory effects [32],[34],[35]. Finally, to accommodate experimental evidence that several neural levels contribute to binocular rivalry, two recent models [45],[50] postulate a feedforward hierarchy of competing levels.

## Models

Our model is stochastic and follows the activity of many independent neural populations. Each population is assumed to possess two stable states - an ‘inactive’ state of low activity and an ‘active’ state of high activity - and to transition back and forth between these states under the influence of input and noise. Transitions are assumed to occur with certain rates (probabilities per unit time), which in turn will be seen to depend on visual input and on the phenomenal percept.

**Figure 1:**
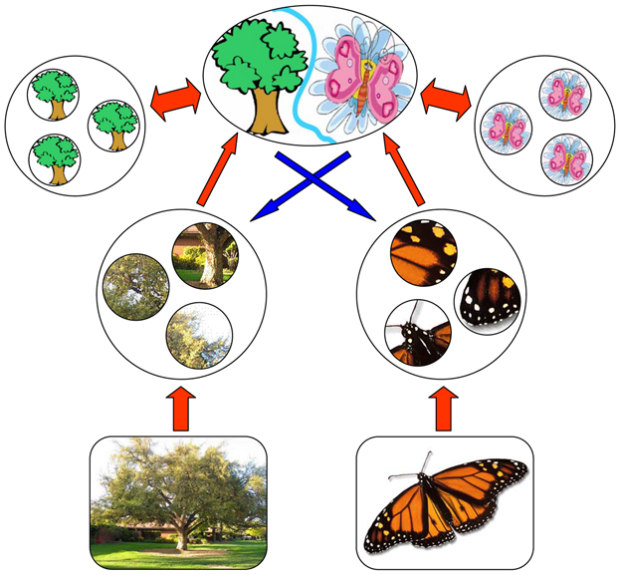
Model architecture for binocular rivalry between two images (‘tree’ and ‘butterfly’). Two types of meta-stable populations – evidence populations (EPs) and memory populations (MPs) – transition independently between ‘inactive’ and ‘active’ states. The evolution of activity in each pool is governed by transition rates. Each percept is associated with one pool of EPs and another pool of MPs. Perceptual dominance depends on the combined activity of the associated EPs and MPs. The colored arrows represent ‘effective’ interactions (excitatory, red; inhibitory, blue) that modulate transition rates. The interdependence of transition rates and combined activity produces periodic reversals of phenomenal experience.

The model postulates two representational levels, one level of ‘evidence populations’ (EPs), which integrate visual inputs over short time-scales, and another level of ‘memory populations’ (MPs), which integrate phenomenal states over longer time-scales. To model the dynamics of binocular rivalry, where there are two possible phenomenal states, we assume two pools of EPs (each with 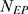 populations) and two pools of MPs (each with 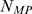 populations), associating each pool with a different phenomenal state. The four pools and their interactions are shown schematically in Figure 1.

For a pool 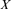 (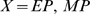) with 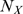 populations, 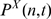 denotes the probability that 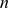 populations are ‘active’ at time 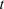, while the 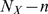 remaining populations are ‘inactive’. Further, 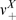 denotes the rate of the inactive→active transition and 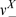 that of the active→inactive transition. We assume that, in the time interval 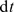, at most one transition can occur, independently of any previous transitions (Poisson process).

Several transition events contribute to the total change 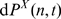 over 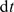. Negative contributions are occasioned by one of 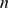 active populations becoming inactive 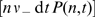, or by one of 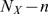 inactive populations becoming active 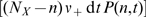. Positive contributions arise from one of 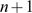 active populations becoming inactive 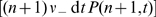, or from one of 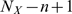 inactive populations becoming active 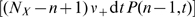


All four contributions enter into the dynamic equation of pool 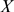:
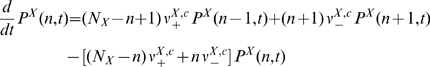
(1)


Here, the superscript 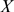 denotes the four pools (evidence and memory populations for two percepts) and the superscript 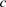 indicates different transition rates (see below). As long as transition rates remain unchanged, the *average* number of active populations in a generic pool approaches the asymptotic value 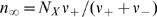 with a characteristic time 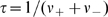. The *asymptotic* number of active populations is a binomially distributed random variable:
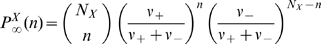
(2)


The phenomenal state ( *i.e.*, the currently dominant percept) is not represented explicitly in the model. Instead, the EPs and MP s associated with each percept are combined and their total number is compared with a threshold 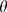. Whenever this number comes to exceed the threshold and the stimulus is on, the associated percept is deemed to gain dominance (even when the other percept's total activity also exceeds 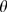 at this moment of time). Once gained, dominance is lost only when a percept's total activity drops below threshold, or when the total activity of the other percept crosses the threshold, too.

An essential aspect of the model is the choice of transition rates. We use transition rates to compactly represent the combined influence of feedforward input (*i.e.*, visual stimulation), of recurrent input, and of the phenomenal percept. In developing the model, we realized that a handful of conditions, each with different transition rates, suffices to generate the rich dynamical behavior of bistable perception. Specifically, we assume an ‘excitation’ of EPs by the stimulus, an additional, ‘selective excitation’ of EPs and MPs associated with the active percept, and a ‘selective inhibition’ of EPs associated with the other percept.

**Figure 2:**
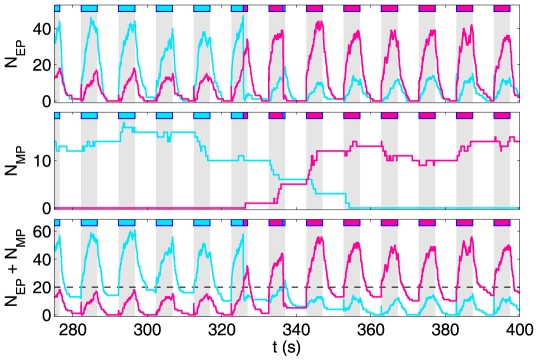
Activity dynamics during the intermittent presentation of a rivalrous display. The three graphs represent the evolution of EP activity (upper), MP activity (middle), and combined activity (lower). In each graph, the activities associated with the two percepts are shown as magenta and cyan curves, respectively. When the combined activity of one percept crosses a threshold (black line in the bottom graph), that percept dominates phenomenal experience (as indicated at the top of each graph by magenta or cyan stripes). Stimulation periods of 4.4 s (grey stripes) alternate with blank periods of 5.7 s. See text for a detailed description of the model dynamics.

Figure 2 illustrates the typical evolution of activity in the different pools, and the resulting perceptual alternations, when a bistable stimulus is periodically interrupted by blank periods. The dynamic evolution distinguishes 4 conditions, depending on the presence or absence of a stimulus and a dominant perceptual state:

**Condition 1:** After stimulus onset, but before a dominant percept has emerged. When a stimulus is present, but no dominant percept has yet emerged, the activity of EPs grows rapidly, mimicking ‘excitation’ by the visual stimulus (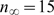, 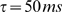). Any activity of MP s decays (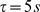).

**Condition 2:** The first 200 ms after one percept (e.g., the ‘butterfly’) has gained dominance. When one percept becomes dominant (because the combined activity of its associated populations exceeds threshold), the now dominant EPs continue to charge, but with longer characteristic times (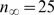, 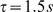), whereas the now suppressed EPs discharge (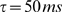). This short-lasting condition stabilizes the newly dominant percept and mimics a ‘transient suppression’ of the EPs associated with the other percept. In effect, this cross-inhibition implements a transient interaction between the active percept and the EPs associated with the other percept. Note that dominance is gained always by the *most recent* percept to cross 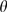. The rapid sequence corresponding to Condition 1 and Condition 2 explains the ‘spikes’ that are sometimes observed (in Figure 2) when stimulation resumes at the end of a blank period.

**Condition 3:** Continued dominance of the same percept. After the brief transition period, the EPs of the dominant percept continue to charge as before, but the EPs of the suppressed percept are now charging as well, albeit more slowly (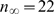, 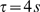). This condition mimics the combined effects of a ‘sustained inhibition’ by the phenomenal percept and an ‘excitation’ by the visual stimulus (see *(1)* above).

In addition to inhibiting EPs, the phenomenal state also excites MPs. Specifically, we assume that the MP s associated with the dominant percept charge slowly, (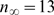, 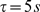, whereas the MP s associated with the suppressed percept discharge at the same rate. This ensures that the phenomenally dominant percept charges its associated memory while discharging the memory of the alternative percept.

**Condition 2′:** The first 200 ms after a reversal, in which the other percept (*e.g.*, the ‘tree’) has gained dominance. This condition is symmetric to Condition 2.

**Condition 3′:** Continued dominance of the ‘tree’ percept (symmetric to Condition 3).

**Condition 4:** Blank display. In the absence of a stimulus, any residual activity dissipates and both EPs and MPs become inactive (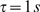 and 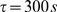, respectively). The rates for MP s are characteristic times for the spontaneous decay of a percept-specific working-memory.

These assumptions (7 integration parameters for EPs, 3 integration parameters for MPs, pool sizes 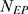 and 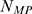) suffice to emulate a large body of empirical observations on the perceptual dynamics of continuous and intermittent displays. Moreover, the predicted behavior is robust over a considerable range of parameter values.

The interaction between total activity in EPs plus MPs and transition rates in EPs and MP s, combined with the stochastic activity dynamics in the four pools, produces an irregular sequence of phenomenal reversals that may be compared directly to experimental observations.

## Results

### Mean dominance times

The main evidence for a memory in bistable perception is the tendency of a percept to persist when stimulation is interrupted: before and after an interruption of stimulation, the subjective appearances are often the same. This persistence slows and perhaps even arrests perceptual reversals in intermittently presented displays [22]–[24],[51]. In our model, the persistence of appearance arises from the existence of memory populations that influence perceptual dominance.

We define the dominance time 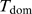 of a percept as the total stimulated time between two reversals. In the case of continuous stimulation, this is simply the time between reversals. In the case of intermittent stimulation, it is the total time minus any blank periods.

**Figure 3:**
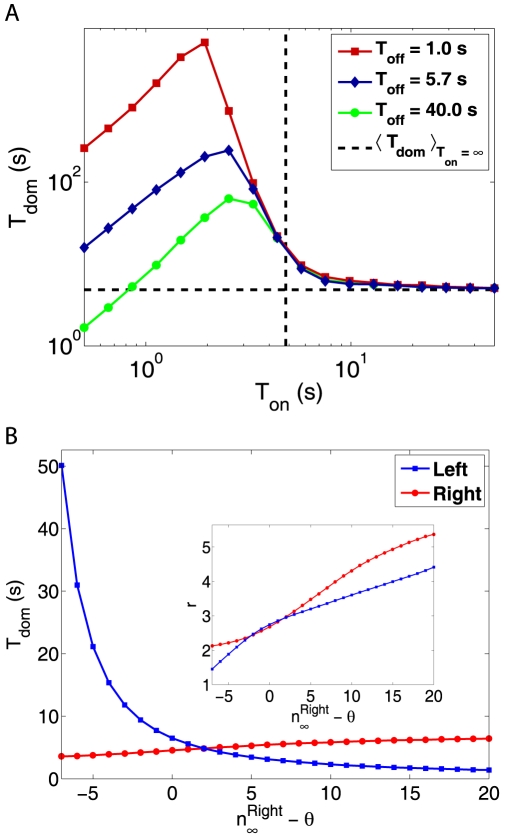
Mean dominance times under interrupted and continuous stimulation. A: Mean dominance times 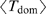 as a function of stimulus period 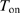, for different blank periods 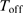. B: Effect of differential stimulus intensity. Dominance times 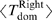 and 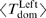 as a function of 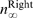, when 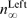 is held constant. The inset shows the corresponding shape parameters 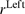 and 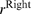 as a function of 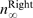.

Our model predicts a complex dependence of the mean dominance time 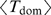 on the stimulation period 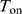 and the blank period 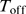 (Figure 3A). Starting from 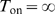 (continuous display), 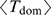 rises slowly from the baseline 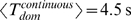 (dashed black lines), the increase becoming dramatic in the proximity of 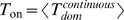. At this point, MP s are maximally active and stabilize phenomenal experience. If perceptual reversals occur at all, they happen at the beginning of, rather than during 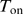. For even smaller 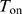, phenomenal experience remains stable for a certain number of display cycles (see **Perceptual persistence**), and 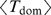 decreases trivially with 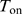. The height and position of the peak in 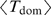 depends also on 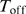, for the average activity of MP s (and, thus, their stabilizing effect) depends on the balance between 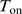 and 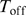.

These predictions account qualitatively for the observation that intermittent stimulation slows perceptual reversals [22]–[24]. Especially for short 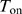, it is known that dominance times grow very long and that perceptual reversals essentially cease [23]. Unsurprisingly, our model fails to predict the behaviour observed for short 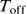 (<1 s) [52], which is thought to reflect fast adaptation.

Raising stimulus intensity (*i.e.*, luminance and/or color contrast) can be assumed to monotonically increase the parameter 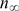. When left- and right-eye images present different intensities, the evidence populations associated with the left- and right-image EPs will exhibit different parameter values, 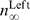 and 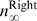, respectively.

It is interesting to explore how different choices of 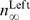 and 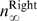 affect the perception of a continuous display. When (say) 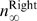 is increased while 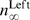 is held constant, dominance times increase slightly for the right image but decrease dramatically for the left image (Figure 3B). When 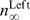 is decreased, the intersection in Figure 3B shifts to the left (not shown), as reported by [17]. This confirms that 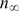 is a plausible substitute for stimulus intensity.

The qualitative behavior in Figure 3B is empirically well established and is known as “Levelt's second proposition” [5],[17]. The reason for this behavior is that, in our model, reversals are triggered by the charging of the suppressed percept. As charging rate increases with stimulus intensity (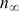), greater stimulation of the suppressed percept shortens 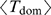 for the dominant percept.

### Distribution of dominance times

Dominance times of both human and non-human observers in binocular rivalry and other types of bistable displays exhibit a Gamma-like distribution 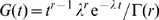, where 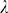 is a rate constant and 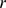 is a shape parameter. The mean dominance time is 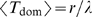 and the coefficient of variation of dominance times is 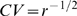. Empirically, rate 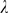 and mean time 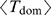 range over almost two OM, whereas the shape parameter 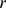 is largely preserved and varies only by half an OM [30],[31]. One important aim of our model is to account for this uncoupling of the shape parameter 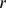 from the mean time 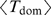.

In our model, perceptual reversals reflect the rapid accumulation of stimulus evidence below the perceptual threshold by evidence populations (EPs). Only three parameters matter for the distribution of dominance times, namely, the total number of evidence populations, 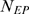, the number of active evidence populations at equilibrium, 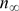, relative to the perceptual threshold 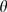, and the relaxation time 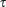. Of these three, the parameter 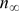, which represents stimulus intensity, proves the most consequential.

**Figure 4:**
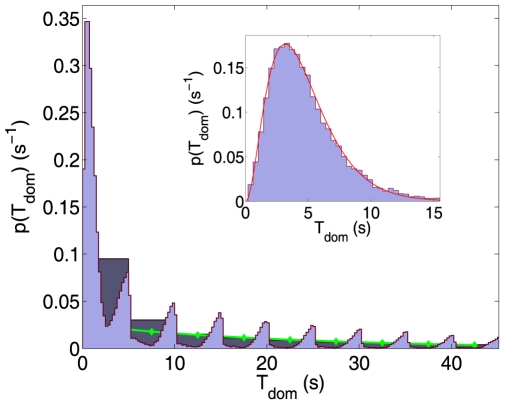
Distribution of 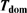 for intermittent display with 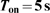, 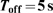, and 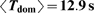. Darker bins in the background: integral probability of a perceptual switch between the 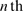 and the 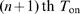; for 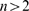, the histogram is well approximated by an exponential (continuous line: best exponential fit for 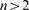). Inset: distribution of 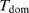 for continuous display. Blue bars: histogram of 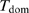 from simulations (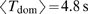), red line: fitted Gamma-distribution, with 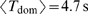 and 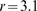.

For continuous displays, our model replicates a Gamma-like distribution of dominance times for a wide range of parameter choices (see inset in Figure 4). Intuitively, this may be understood as follows: if 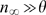, EP+MP crosses the threshold almost deterministically, resulting in a Gaussian distribution of dominance times (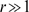). On the other hand, if 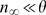, EP+MP will cross the threshold only in the event of rare fluctuations, producing an exponential distribution of dominance times (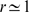). Intermediate situations with 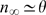, lead to Gamma-like distributions with 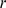 ranging from 3 to 6.

For example, in Figure 3B, the shape parameter 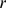 varies in a comparatively narrow range (see inset), whilst the ratio of 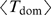 s varies over almost two orders of magnitude. Note that the ‘left’ values of 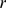 and 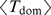 exhibit strongly opposing trends. This marked anti-correlation is a sign of the stochastic mechanism for threshold crossing: with lower stimulus intensity 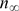, threshold crossings become rarer and the interval distribution becomes more Poisson-like.

Note also the (slight) positive correlation between the ‘right’ values of 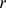 and 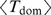 in the inset of Figure 3B (red curve). This constitutes a prediction that depends strictly on memory effects and that goes beyond “Levelt's second proposition” [5]. To understand this positive correlation, consider a situation where integration is driven by fluctuations and times-to-threshold are comparatively long and exhibit Poisson-like statistics (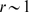). In this situation, the shape parameter 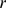 reflects the number of Poisson-like ‘jumps’ that are required to reach threshold 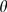. The primary consequences of an increase in 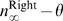 are that ‘left’ dominance times decrease sharply while ‘right’ dominance times increase slightly. As a secondary consequence, the ‘left’ memory activity also decreases, which raises the number of ‘jumps’ required by the ‘left’ integration and thus also the ‘right’ value of 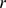. This accounts for the parallel trends in the ‘right’ values of 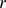 and 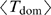.

In general, when the stimulus intensity 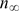 is varied either in one eye or in both, our model makes a qualitative prediction for the average dominance distribution (comprising dominance times of both percepts): the average values of 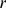 and 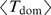 should be anti-correlated. Interestingly, there seems to be some evidence for such a trend [31].

For intermittent displays (Figure 4, 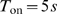, 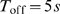), our model predicts a multi-peaked distribution: the integral probability of a perceptual switch between the 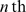 and the 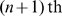 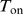 (darker bins in the background), for 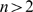, is well approximated by an exponential (continuous line: best exponential fit for 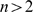). The spikes in the distribution reflect the periodicity of the stimulation and are separated roughly by 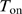. They comprise the probability of a perceptual switch at the onset and during continued presentation. Assuming that the MPs of the current winning percept have reached a stationary state, both these probabilities do not vary statistically from one 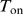 to the next, leading to an exponential decay for large enough 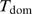 (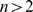, or twice the characteristic time of MPs). During the first two 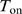, the MP s are still charging after the last perceptual switch and a perceptual reversal is more likely than for 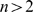. The first anomalous peak in the distribution is attributable to the very brief dominance intervals that usually occur during periods of ‘uncertainty’, when the level of the MP s is roughly equal for both percepts (see the central part of Figure 2 for an illustration).

There are few empirical reports of dominance distributions for intermittent displays. Both Gamma-shaped [37] and monotonically decreasing [51] distributions have been reported. However, further experiments are needed to establish the generality of these results

### Sequential correlations

Successive dominance intervals in bistable perception are thought to be statistically almost independent [25],[26]. This is why bistable perception was long considered a “memoryless” process [25],[27],[28],[31].

**Figure 5:**
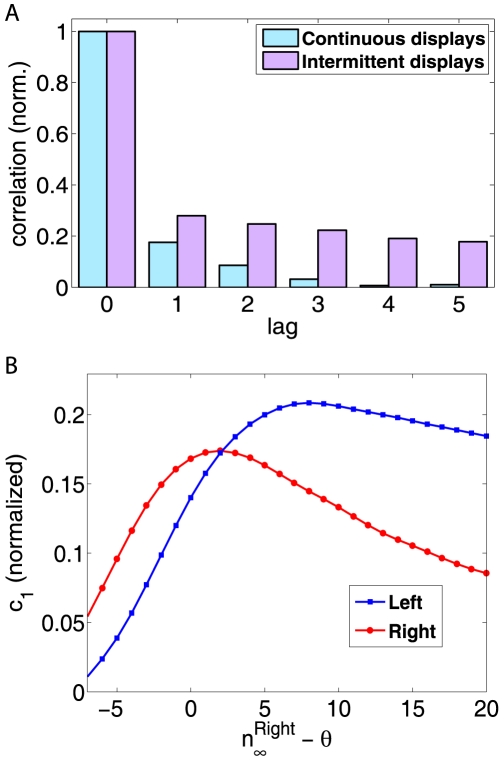
Sequential correlations for continuous and intermittent displays. A: Correlation coefficient 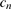 between dominance periods 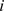 and 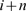, as a function of 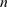 and normalized to 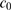. B: Effect of differential stimulus intensity of continuous display. Correlation coefficient 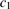 and 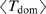 of both percepts, computed for different values of 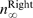. Data are from the same simulations as in Figure 3B.

However, the existence of memory representations predicts small but significant departures from sequential independence. Figure 5A shows the predicted correlation between a given dominance period and its 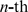 successor. Interestingly, the predictions differ for continuous and intermittent presentation.

Figure 5B shows the correlation (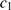) between successive dominance periods of percept ‘Left’ (blue) and percept ‘Right’ (red), for continuous presentations, as functions of 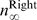 (same simulations as in Figure 3B).

The non–monotonic behaviour observed is another consequence of MP dynamics. When one of the 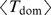 is much larger than the characteristic times of MP s (left part of the plot), the activity level of MP s is essentially constant (either low or high) and cannot provide correlation effects; if the average 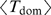 is much smaller than the characteristic times of MP s, memory effects do not have time to build up and again cannot sustain correlations (right part of the plot). Finally, whenever the distribution of dominance times becomes narrow (high 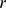 values), so that the variance is inherently small, sequential correlations will be negligible.

Taken together, Figure 3B and Figure 5B suggest that an experimental verification of Levelt's second proposition should reveal specific links between 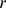, 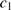 and 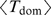 that result, at bottom, from memory effects.

For continuous displays, correlations are largest for intermediate values of stimulus intensity, when MP s charge partially and the degree of charging varies from time to time (Figure 5B).

The peak position reflects the characteristic times of the MP s (about 5 s). For other values of 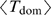, the charging is either to little or too complete to produce large correlations.

Memory-induced correlations should be somewhat larger in intermittent displays, as the normal alternation of dominant percepts is suspended and the same percept dominates for several successive display intervals. In this situation, the differential activity between the MP s of dominant and suppressed percepts grows larger and stochastic fluctuations in this difference induce more noticeable correlations (Figure 5A).

### Perceptual persistence

**Figure 6:**
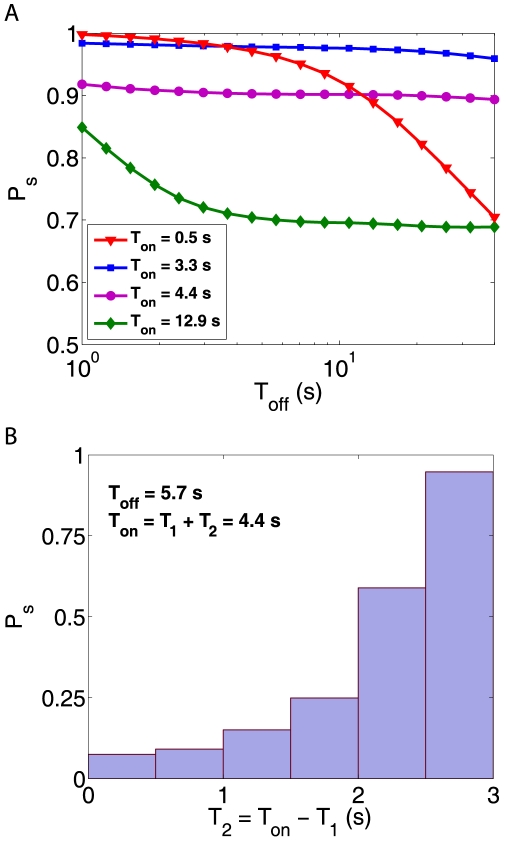
Survival probability 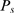 and perceptual history. A: Joint dependence on 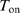 and 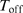, see text for details. B: When 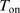 contains two dominance phases of durations 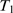 and 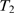, 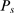 decreases with 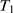 (less recent phase) and increases with 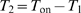 (more recent phase).

In intermitted displays, the persistence of a percept across the stimulation gap is often measured in terms of a ‘survival probability’ 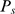 [23], *viz.* the probability of the same percept dominating before and after the gap. Our model predicts an interesting and complex dependence of 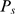 on stimulus duration 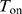 and blank duration 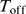, which is illustrated in Figure 6A.

For short 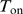, the MP s do not charge and the survival probability 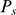 is influenced only by differential activity in the EPs, which decays rapidly after stimulus termination. For this reason, 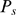 decreases rapidly with increasing 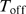 (Figure 6A, red curve). When 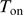 is long enough to charge MP s, but too short to permit spontaneous reversals, 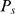 is governed by memory and remains close to unity as long as the memory persists (Figure 6A, purple and blue curves). Finally, when 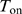 is long enough to permit spontaneous reversals, the memory activity of both percepts is comparable and 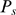 reflects differential activity in the EPs (Figure 6A, green curve).

Some of these predictions are borne out by published evidence. For example, Leopold and colleagues reported uniformly high 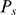 for intermediate values of 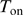 (400 ms; [23]). For longer 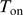 that permitted spontaneous reversals, survival probability 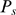 progressively decreased.

When 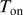 permits two dominance periods, survival probability 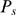 reflects the relative durations [23],[32],[33]: 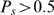 when the most recent period lasted longer than the less recent period and 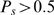 when the situation was reversed. Our model readily accounts for these observations (Figure 6B), provided 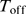 is sufficiently large. The regime of 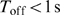 [34],[52],[53], where fast adaptation could become important, is again out of the scope of our model.

## Discussion

We propose that binocular rivalry, and other instances of bistable perception, reflect the stochastic integration of many meta-stable populations at two levels of neural representation, *viz.* sensory input and perceptual experience. While previous accounts of bistable perception rely on an oscillatory dynamic, our model is inherently stochastic. We argue that a fluctuation-driven process accounts naturally for key characteristics of bistable perception that have remained puzzling for decades.

One of these puzzling characteristics is the wide range of average times between perceptual reversals, which for different observers, display types, and stimulus properties can extend over two orders of magnitude [30],[31]. Another unexplained finding is the preserved stochasticy of reversals, that is, the fact that the statistical distribution of times between reversals is Gamma-like and exhibits a shape parameter 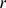 with typical values from 3 to 6.

Taken together, these observations strongly suggest a fluctuation-driven escape process. In such a process, the system state fluctuates until it reaches an escape threshold, at which point it is reset some distance away from threshold. Depending on the asymptotic value of the integration process, the average frequency of threshold crossings can vary over more than one order of magnitude, while the distribution of times between threshold crossings will retain its Gamma-like shape. This uncoupling of mean dominance time and shape parameter is an important advance over previous models and is illustrated in Figure 3B.

Following this general insight, we model bistable perception as a ‘race’ between two independent processes of stochastic integration, each concerning multiple neuronal pools that are individually meta-stable between inactive and active states. We further assume an escape threshold and a competitive reset mechanism that resets each process whenever the other process reaches threshold.

Previous models of bistable perception postulate a deterministic process at the level of individual neurons (i.e., spike-frequency adaptation [32],[34],[35],[54] or synaptic depression [44]–[46]) which drives the system towards a reversal threshold. The resulting oscillatory dynamic is typically perturbed by a suitable level of neural noise [17], [35], [47]–[49]. In such an ‘oscillator model’, the average time between reversals is set by thedeterministic process while the statistical distribution of these times directly reflects the level of noise. For a given set of parameters, oscillator models such as [32],[35] produce either a realistic, Gamma-like distribution of dominance times or a realistic dependence of mean dominance times on stimulus properties (*e.g.*, intensity or timing), but not both. For example, an oscillator model such as [35] accounts for the dependence of dominance times on stimulus times only in the absence of noise. When the model is imbued with realistic levels of noise (so that 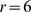), the dependence on stimulus intensity all but disappears.

Yet another puzzling characteristic of bistable perception is the hysteresis or memory effects that become evident when visual presentation is interrupted [23],[24]. To summarize the available evidence, the history of percepts prior to an interruption biases perception once stimulation resumes. Memory effects are long-lasting and are characterized by time-scales an order of magnitude larger than those of perceptual reversals [23],[33]. Memory effects are stabilizing in that they favor the recurrence of percepts that have dominated already in the past. Not only the most recent percept, but also less recent percepts that have dominated longer, leave a measurable bias [23],[32],[33]. Finally, the stabilizing influence of perceptual history is evident not only in the percept that dominates a renewed stimulus onset but also in the duration of dominance phases following that onset [55].

To account for memory effects, several oscillator models have been extended to include an additional interaction or state variable [32],[34],[35]. However, none of these models captures the entire range of experimental findings. The model of Noest and colleagues [34] lacks a second, longer time-scale and does not account for observations with long interruptions of stimulation. The models of Wilson [35] and of Brascamp and colleagues [32] include multiple time-scales and do capture long-lasting memory effects. However, the Wilson model [35] does not account for the influence of the duration of dominance phases preceding the stimulus interruption [23],[32],[33]. Conversely, the model of Brascamp and colleagues [32] fails to predict the observed effect on dominance durations following the stimulus interruption [55].

Our stochastic-integration model incorporates two time-scales in the form of ‘evidence populations’ (EPs with higher transition rates) and ‘memory populations’ ( MP s with lower rates). A material difference to other models [32],[35] is that EPs are driven by sensory evidence and perceptual state, while MP s are driven only by perceptual state. This ensures that the memory of a perceptual state builds up while this state persists and correctly predicts all effects *of* and *on* dominance duration that have been reported so far [23],[32],[33],[55]. The recurrent influence of perceptual state on both MP s and EPs distinguishes our model from other two-level models [45],[50], which employ a strictly feedforward architecture.

With one major exception (see below), our model comprehensively predicts the dynamics of bistable perception for continuous and intermittent displays. For example, it predicts dominance times, dominance distribution shape, sequential correlations between dominance times, and perceptual persistence across blank periods, including, in the case of intermittent displays, the dependence of these quantities on 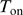 and 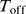. Some of the predictions bear out past experimental observations: the degree to which phenomenal experience is stabilized with different values of 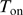 and 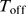 in an intermittent display [22]–[24], or the dependence of phenomenal experience on a history comprising several preceding dominance periods [23],[32],[33]. Several other predictions of interest are yet to be tested, however. For example, our model predicts how the shape of the dominance distribution (Figure 4) and the size of sequential correlations (Figure 5) should vary with 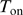 and 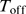 under conditions of intermittent presentation.

An important test for models of bistable perception are the opposite and unequal changes in dominance time that results from an asymmetric changes in stimulus intensity (“Levelt's second proposition”) [5]. Our model correctly predicts the unequal dependence of dominance times on the intensity of a *weaker* stimulus and partially predicts the *reversed* dependence of dominance times on the intensity of a *stronger* stimulus [17].

In its current form, our model does not account for the well-known effects of visual adaptation [39], [56]–[60] on bistable perception. This omission is intentional and is meant to highlight the dynamic possibilities offered by stochastic integration on the longer time-scales at which adaptation effects are expected to be small. The absence of adaptation implies that our model cannot account for the phenomenon of “flash suppression” [61],[62] and, more generally, for the perceptual effects of brief stimulus interruptions (<1000 ms) [22],[34],[52],[53].

For the sake of simplicity, our model is formulated in terms of abstract, meta-stable populations governed by transition probabilities. The underlying idea is that each population represents a recurrently connected network of spiking neurons, with two metastable attractor states [63]–[67]. In such a ‘working-memory-type’ network, stochastic transitions between attractor states are driven by internally generated fluctuations in network activity [49], [65], [68]–[71]. The transition probabilities 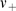 and 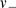 are the escape rates from the two attractor states: the lower the attraction force, the higher the escape rate. Importantly, the transition rates depend less on the time-constants of individual neurons than on the average activity level and the amplitude of activity fluctuations in relation to the transition threshold. This is why small differences in recurrent connectivity can shift transition rates by some orders of magnitude [68]–[70].

Our model postulates that perceptual dominance reflects a collective decision on the basis of two distributed representations (*viz.*, two pools of meta-stable populations). The stochastic integration of those representations provides the accumulated information for the perceptual decision; such a mechanism has been also proposed as a substrate for the perception of time [71],[72]. In a detailed (spiking network) model, such a collective decision would require convergent synaptic projections to a readout stage, where competitive interactions could ensure that any decision is categorical [73],[74]. In other words, our model predicts the existence of a competitive stage receiving projections from all evidence and memory populations. This hypothetical stage would somewhat resemble the “saliency map” that has been postulated by some authors [75],[76].

Finally, excitatory and inhibitory projections between representational (evidence and memory populations) and readout levels could generate the facilitatory and suppressive interactions that are needed to start the stochastic integration process over and over again. Such competitive- cooperative interactions in a multi-level network have been studied in the context of visual attention modeling [77].

In conclusion, we suggest that bistable perception is a fluctuation-driven process and is best understood in terms of a progressive integration of, and a collective competition between, ‘working-memory-type’ populations at multiple neural levels.
